# Traumatic Duodenal Rupture and Avulsion of the Ampulla of Vater

**DOI:** 10.1155/1994/12435

**Published:** 1994

**Authors:** M. R. Cox, M. C. Eastman

**Affiliations:** 1 Department of Surgery Goulburn Valley Base Hospital Shepparton Victoria Australia; 2 Department of Surgery Flinders Medical Centre Bedford Park 5042 Australia

## Abstract

Duodenal injury following blunt abdominal trauma is uncommon. The severity of injury can vary from an intramural haematoma to a duodenal rupture with associated transection of the pancreatic duct. A case of duodenal rupture with avulsion of the ampulla of Vater is presented and discussed.


*HPB Surgery*, 1994, Vol. 7, pp. 225–229
Reprints available directly from the publisher
Photocopying permitted by license only

© 1994 Harwood Academic Publishers GmbH
Printed in the United States of America

### CASE REPORT

### TRAUMATIC DUODENAL RUPTURE AND AVULSION OF THE AMPULLA OF VATER

M.R. COX and M.C. EASTMAN
*Department of Surgery, Goulburn Valley Base Hospital, Shepparton, Victoria, Australia*

*(Received 14 December 1992)*

Duodenal injury following blunt abdominal trauma is uncommon. The severity of injury can vary from an intramural haematoma to a duodenal rupture with associated transection of the pancreatic duct. A case of duodenal rupture with avulsion of the ampulla of Vater is presented and discussed.

KEY WORDS: Duodenal rupture, duodenal trauma, pancreatic trauma

### INTRODUCTION

Duodenal injury following blunt abdominal trauma is uncommon, occurring in only 3.4 to 5% of patients with blunt abdominal trauma[1,2]. It is notoriously difficult to diagnose[1,3,4,5]. Delayed diagnosis and treatment is associated with a higher mortality and morbidity[5,6,7]. Duodenal injuries are classified into 4 types[5]: Type 1: a duodenal haematoma or serosal tear. Type 2: a full thickness disruption of the duodenal wall without any associated pancreatic trauma. Type 3a: disruption of the duodenum with a pancreatic haematoma or Type 3b: a duodenal haematoma with pancreatic duct transection. Type 4 is a combined disruption of both duodenum and pancreatic duct. We report a case of duodenal rupture with avulsion of the ampulla of Vater which is best classified as a Type 3c duodenal injury. Only five cases have been previously reported[8-12].

### CASE REPORT

A fourteen year old school boy presented to the Emergency Department of the Goulburn Valley Base Hospital in October 1988, half an hour after sustaining blunt abdominal trauma by running into the cross bar of an upturned hockey goal. He complained of epigastric and right upper quadrant pain. Examination revealed he was afebrile, pulse was 84 and was normotensive. Abdominal findings revealed no external bruising but tenderness in the right upper quadrant extending toward the right iliac fossa. Electrolytes, urea, haemoglobin, white cell count and serum

Address correspondence to: Dr Michael R. Cox, Department of Surgery, Flinders Medical Centre, Bedford Park 5042, South Australia amylase were all normal. A plain abdominal X-ray (Figure 1) revealed free retroperitoneal gas.

The patient was commenced on intravenous cefoxitin and flagyl and an urgent laparotomy was performed. At operation there was a bile stained, retroperitoneal haematoma extending from the second part of the duodenum toward the caecum.

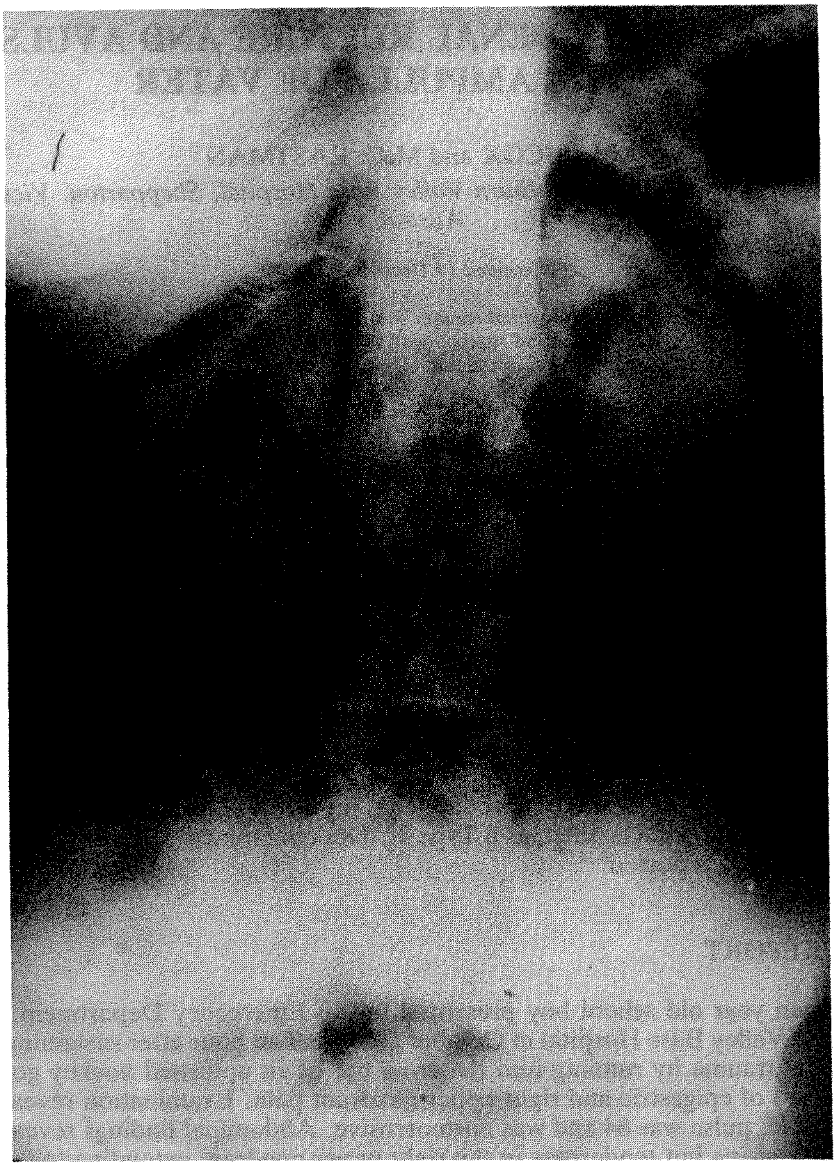

**Figure 1** The plain abdominal X-ray at the time of admission showing extensive retroperitoneal gas.

The duodenum was mobilised to reveal a 75% transverse rupture of the second part of the duodenum. The rupture extended from the posterior wall, around the lateral wall onto the anterior wall. The medial duodenal wall was intact. Closer inspection of the pancreatic head revealed that the ampulla of Vater had been avulsed from the external duodenal wall (Figure 2A). There were no other associated biliary, hepatic, gastric, splenic, colonic or small bowel injuries.

The edges of the duodenal rupture were debrided and repaired with an end to end duodenoduodenal anastomosis. After performing a cholecystectomy, the lower end of the common bile duct (CBD) was transected and a choledochoduodenostomy was performed. The distal CBD was over sewn. The head of the pancreas was mobilised away from the medial duodenal wall to allow it to lie anterior to the repaired duodenum. The transected ampulla was debrided and anastomosed to a Roux-en-Y jejunal loop (Figure 2B). A percutaneous gastromy and feeding jejunostomy were performed and low pressure suction drains were placed adjacent to the duodenal and pancreatic anastomoses.

Post operatively there were no septic complications and no rise in serum amylase. The only complication was persistent gastric outlet obstruction for 5 weeks. From the sixth post operative day nutrition was maintained with jejunostomy feeding. Enteral feeding was continued until the twenty-ninth post operative day, adding 100 ml of the gastric aspirate to every 250 ml of enteral feed to prevent excessive bile salt depletion. On the eighteenth post operative day a barium meal revealed duodenal obstruction and oedema. By the twenty-eighth post operative day the gastric aspirate was reduced to less than 100 ml. A repeat barium meal revealed free flow through the duodenum into the jejunum. Oral nutrition was established by the thirtieth post operative day and the patient was discharged 2 days later. After a 4 year follow up he is well with no symptoms, no evidence of malabsorption and normal liver function tests.

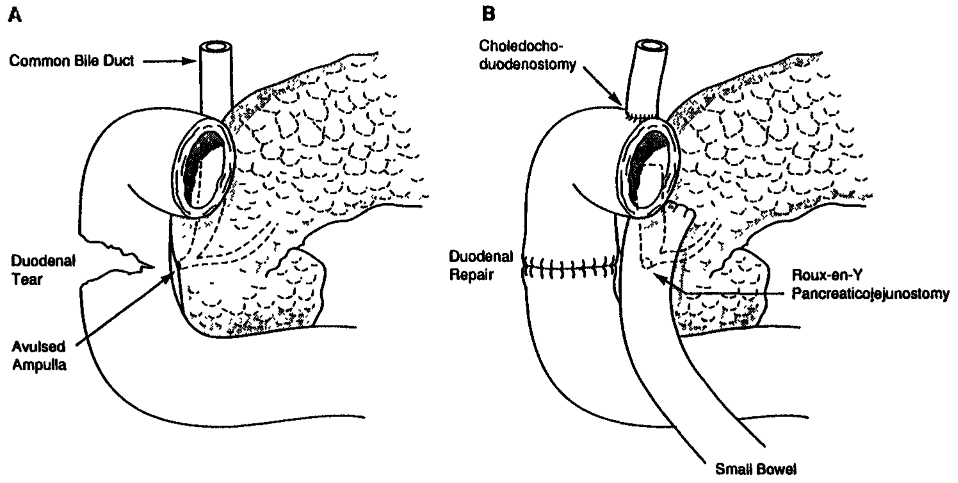

**Figure 2** A: A sketch of the partial transection of the duodenum with an intact medial wall and avulsed ampulla of Vater. B: The operative repair consisting of a primary duodenal closure, choledochoduodenostomy and pancreatico-jejunostomy Roux-en-Y.

### DISCUSSION

Rupture of the duodenum with avulsion of the ampulla of Vater is very rare, with only 5 previous reports in the literature[8-12]. The injury represents a duodenal injury that has not been adequately classified[5]. As it is not a transection of the body of the pancreas and duct, it is best given a separate classification as a Type 3c duodenal injury[3,4,7]. In this and the previous 5 reported cases no resectional surgery was required[8-12], unlike Type 4 injuries that often require a Whipple's operation[3,4,7].

The diagnosis of a ruptured duodenum was easy in this case with gross retroperitoneal air on the plain abdominal X-ray. This is an uncommon finding occurring in only 5% of cases of duodenal rupture[4], with more subtle changes of a blurred psoas shadow or scoliosis to the right occurring in up to 50% of cases[5,13,14]. Serum amylase is elevated in less than 10% of cases of duodenal rupture[4]. The diagnosis of a ruptured duodenum is often difficult and must be suspected in any blunt abdominal trauma. A gastrograffin swallow may be useful when diagnosis is being considered[4,5].

At laparotomy complete mobilisation of the duodenum is essential as the perforation may have a considerable posterior component. In cases with a duodenal rupture the pancreas must be carefully inspected to avoid overlooking pancreatic transection or an avulsion of the ampulla. The duodenal rupture can usually be closed primarily with careful debridement and an end-to-end anastomosis[5,6,8-11]. Adequate defunctioning of the duodenum is required and in our case was achieved with a gastrotomy tube. Other authors use diverting techniques such as "duodenal diverticularisation" where the first part of the duodenum is transected and gastroenterostomy performed[15] or "pyloric exclusion" where the pylorus is over sewn from within via a gastrotomy and then a gastroenterostomy performed using the gastrotomy site[16,17]. A simple gastrostomy was performed as it adequately drains the stomach, is simple, less time consuming, retains the normal anatomy and is associated with a low incidence of duodenal leakage and subsequent septic episodes or duodenal fistula[4,5,6]. As return of gastric emptying is often delayed, the gastrotomy tube avoids the complications of prolonged nasogastric intubation such as nasal ulceration, oeosophagitis and subsequent stricture and improves the post operative respiratory care of the patient.

Four of the previous five reports with avulsion of the ampulla have performed a primary duodenal repair with re-implantation of the ampulla[8-11]. This was not performed in the case presented for several reasons. Firstly, there was concern that pancreatitis may occur as a result of the mixing of bile and pancreatic secretions, as occurred in two previous reports[9,11]. Secondly, the viability of the distal end of the ampulla was in doubt and once debrided, the long term patency of the bile duct could not be assured. Thirdly, due to duodenal shortening following the duodenal repair, the head of the pancreas and ampulla would not rest neatly within the duodenal "C", tending to prolapse anteriorly. This made a pancreaticojejunostomy Roux-en-Y technically more easy to perform.

Nutritional support is a problem that must be considered early following duodenal trauma as most patients need prolonged nutritional support, for an average of 3 weeks[2,4,6,7,15]. Many authors rely on total parenteral nutrition (TPN). However, TPN is associated with complications of central line insertion and maintenance, is more expensive[18] and may delay gastric emptying[19]. An alternative method of nutrition is jejunal feeding. This avoids the problems associated with

TPN. Jejunal feeding may be complicated by feeding evoked diarrhoea and abdominal cramps which can usually be controlled with reduction in the flow rate[20]. Another advantage of enteral feeding maintenance of the entero-hepatic metabolism of bile salts.

### *References*

1. Ghuman, S.S., Pathak, V.B., McGovern, P.J., Machiedo, G.W.A.P., Swaminathan, A.P. and Lazaro, E.J. (1982) Management and complications of duodenal injuries. *Am.Surg.*, **48**, 109–113
2. Kelly, G., Norton, L., Moore, C. and Eiseman, B. (1978) The continuing challenge of duodenal injuries. *J.of Trauma*, **18**, 160–165
3. Adkins, R.B. and Keyser, J.E. (1985) Recent experiences with duodenal trauma. *Am.Surg.*, **51**, 121–131
4. Flint, L.M., McCoy, M., Richardson, J.D. and Polk, H.C. (1980) Duodenal injury: Analysis of common misconceptions in diagnosis and treatment. *Ann.Surg.*, **191**, 697–702
5. Lucas, C.E., Ledgerwood, A.M. (1975) Factors influencing outcome after blunt duodenal injury. *J.Trauma*, **15**, 839–846
6. Corley, R.D., Norcross, W.J. and Shoemaker, W.C. (1975) Traumatic injuries of the duodenum: A report of 98 patients. *Ann.Surg.*, **181**, 92–98
7. Snyder, W.H., Wegelt, J.A., Watkins, W.L. and Bieta, D.S. (1980) The surgical management of duodenal trauma: Precepts based on a review of 247 cases. *Arch.Surg.*, **115**, 422–430
8. Cooke, H.S. (1990) Avulsion of the ampulla of Vater following blunt trauma. *Aust.N.Z. J.Surg.*, **60**, 393–396
9. Farelli, G. and Nacchiero, M. (1980) An interesting case of pancreaticoduodenal trauma *Am.J.Gastro.*, **74**, 279–281
10. Parkinson, S.W. and Wisniewski, Z.S. (1978) Avulsion of the Ampulla of Vater: An isolated injury following blunt abdominal trauma. *Aust.N.Z. J.Surg.*, **48**, 562–564
11. Lee, D., Zacker, J. and Vagel, T.T. (1976) Primary repair in transection of duodenum with avulsion of the common duct. *Arch.Surg.*, **111**, 592–593
12. Fish, J.C. and Johnson, G.L. (1966) Rupture of duodenum following blunt trauma: Report of a case with avulsion of papilla of Vater. *Ann.Surg.*, **162**, 917–919
13. Matolo, N.M., Cohen, S.E., Fontanetta, A.P. and Wolfman, E.F. (1975) Traumatic duodenal injuries: An analysis of 32 cases. *Am.Surg.*, **6**, 331–336
14. Fabian, T.C., Mangiante, E.C. and Millis, M. (1984) Duodenal rupture due to blunt trauma: A problem diagnosis. *South Med.J.*, **77**, 1078–1082
15. Berne, C.H., Donovan, A.J. and White, E.J. (1974) Duodenal diverticularisation for duodenal and pancreatic injury. *Am.J.Surg.*, **127**, 503–507
16. Vaughan, C.D., III, Frazier, O.H. and Graham, D.Y. (1977) The use of pyloric exclusion in the management of severe duodenal injuries. *Am.J.Surg.*, **134**, 785–790
17. Martin, T.D., Feliciano, D.V., Mattox, K.L. and Jordon, G.L. (1983) Severe duodenal injuries: Treatment with pyloric exclusion and gastrojejunostomy. *Arch.Surg.*, **118**, 531–535
18. Heylen, A.M., Lybeer, M.B., Penninckx, F.M. and Frost, P.G. (1987) Parental versus needle jejunostomy nutrition after total gastrectomy. *Clin.Nut.*, **6**, 131–136
19. Sheiner, H.J. and Catchpole, B.N. (1976) Drug therapy for post vagotomy gastric stasis. *Br.J.Surg.*, **63**, 608–611
20. Cade, R.J. (1990) Feeding jejunostomy: Is its routine use in major upper gastrointestinal surgery justified? *Aust.N.Z. J.Surg.*, **60**, 621–623

*(Accepted by M.Little 14 December 1992)*

